# Overlap between eQTL and QTL associated with production traits and fertility in dairy cattle

**DOI:** 10.1186/s12864-019-5656-7

**Published:** 2019-04-15

**Authors:** I. van den Berg, B. J. Hayes, A. J. Chamberlain, M. E. Goddard

**Affiliations:** 1 Faculty of Veterinary & Agricultural Science, University of Melbourne, Parkville, Victoria Australia; 2 Agriculture Victoria, AgriBio, Centre for AgriBioscience, Bundoora, Victoria Australia; 3 Queensland Alliance for Agriculture and Food Innovation, Centre for Animal Science, University of Queensland, St Lucia, Queensland 4067 Australia

**Keywords:** eQTL, QTL, Dairy cattle, Quantitative traits, Gene expression

## Abstract

**Background:**

Identifying causative mutations or genes through which quantitative trait loci (QTL) act has proven very difficult. Using information such as gene expression may help to identify genes and mutations underlying QTL. Our objective was to identify regions associated both with production traits or fertility and with gene expression, in dairy cattle. We used three different approaches to discover QTL that are also expression QTL (eQTL): 1) estimate the correlation between local genomic estimated breeding values (GEBV) and gene expression, 2) investigate whether the 300 intervals explaining most genetic variance for a trait contain more eQTL than 300 randomly selected intervals, and 3) a colocalisation analysis. Phenotypes and genotypes up to sequence level of 35,775 dairy bulls and cows were used for QTL mapping, and gene expression and genotypes of 131 cows were used to identify eQTL.

**Results:**

With all three approaches, we identified some overlap between eQTL and QTL, though the majority of QTL in our dataset did not seem to be eQTL. The most significant associations between QTL and eQTL were found for intervals on chromosome 18, where local GEBV for all traits showed a strong association with the expression of the FUK and DDX19B. Intervals whose local GEBV for a trait correlated highly significantly with the expression of a nearby gene explained only a very small part of the genetic variance for that trait. It is likely that part of these correlations were due to linkage disequilibrium (LD) in the interval. While the 300 intervals explaining most genetic variance explained most of the GEBV variance, they contained only slightly more eQTL than 300 randomly selected intervals that explained a minimal portion of the GEBV variance. Furthermore, some variants showed a high colocalisation probability, but this was only the case for few variants.

**Conclusions:**

Several reasons may have contributed to the low level of overlap between QTL and eQTL detected in our study, including a lack of power in the eQTL study and long-range LD making it difficult to separate QTL and eQTL. Furthermore, it may be that eQTL explain only a small fraction of QTL.

**Electronic supplementary material:**

The online version of this article (10.1186/s12864-019-5656-7) contains supplementary material, which is available to authorized users.

## Background

A large number of quantitative trait loci (QTL) has been identified for various traits in dairy cattle [1–5]. However, identifying the causative mutation or gene through which a QTL acts has proven very difficult. This difficulty is largely because of the small effect size of most QTL, extensive long-range linkage disequilibrium (LD) in cattle [6] and our lack of understanding of the mode of action of most QTL. Some QTL are due to changes in the amino acid sequence of a protein but most do not appear to be protein-coding mutations and it is assumed they affect gene regulation in some way. For instance, some QTL might be due to a mutation affecting gene expression, that is they are expression QTL (eQTL). Various studies in human have demonstrated enrichment of eQTL near genome wide association studies (GWAS) hits and colocalisation of eQTL and QTL. For example, Brown et al. [7] found enrichment of small GWAS *p*-values for variants with a high probability to be the causal variant for an eQTL, and detected 47 cases showing evidence of colocalisation between eQTL and GWAS variants for various traits in human. In a study by Nicolae et al. [8], GWAS variants associated with quantitative traits in humans were enriched for eQTL variants, including a 2 fold enrichment of variants associated with Crohn’s disease among eQTL. Zhu et al. [9] found significant enrichment of eQTL for GWAS hits for height, BMI and schizophrenia. Therefore, using gene expression information may help to identify genes and mutations underlying QTL. Littlejohn et al. [10] have recently shown that a major QTL influencing production traits in dairy cattle is also an eQTL related to the expression of *MGST1* and Kemper et al. [11] found a QTL for milk yield that is likely an eQTL for *SLC37A1*. However, in general, few QTL have been convincingly shown to be eQTL for the same reasons that make QTL identification difficult. eQTL and QTL are both common and so, given the long-range LD in cattle, it is likely that an eQTL will be in LD with a QTL and therefore appear associated with the phenotype.

The objective of our study was to identify regions associated both with production traits or fertility and with gene expression, in dairy cattle. QTL can be mapped more accurately by an analysis such as Bayes R than by a conventional one variant at a time GWAS [12, 13]. Because Bayes R fits all variants simultaneously, it is more able to narrow the location of a QTL than a GWAS is. Therefore, in this paper we used the Bayes R results for production traits and fertility, and compare that with gene expression data. It is still difficult to show that an eQTL and a nearby QTL are due to the same causal variant. The colocalisation method is designed to do this by estimating the colocalisation posterior probability (CLPP) [14]. However, it may lose power due to the large number of sequence variants in high LD. Therefore, we used three different approaches to discover QTL that are also eQTL: 1) estimate the correlation between local GEBV and gene expression, 2) investigate whether the 300 intervals explaining most genetic variance for a trait contain more eQTL than 300 randomly selected intervals, and 3) a colocalisation analysis.

## Results

### QTL mapping using GWAS and local GEBV variance

**Fig. 1:**
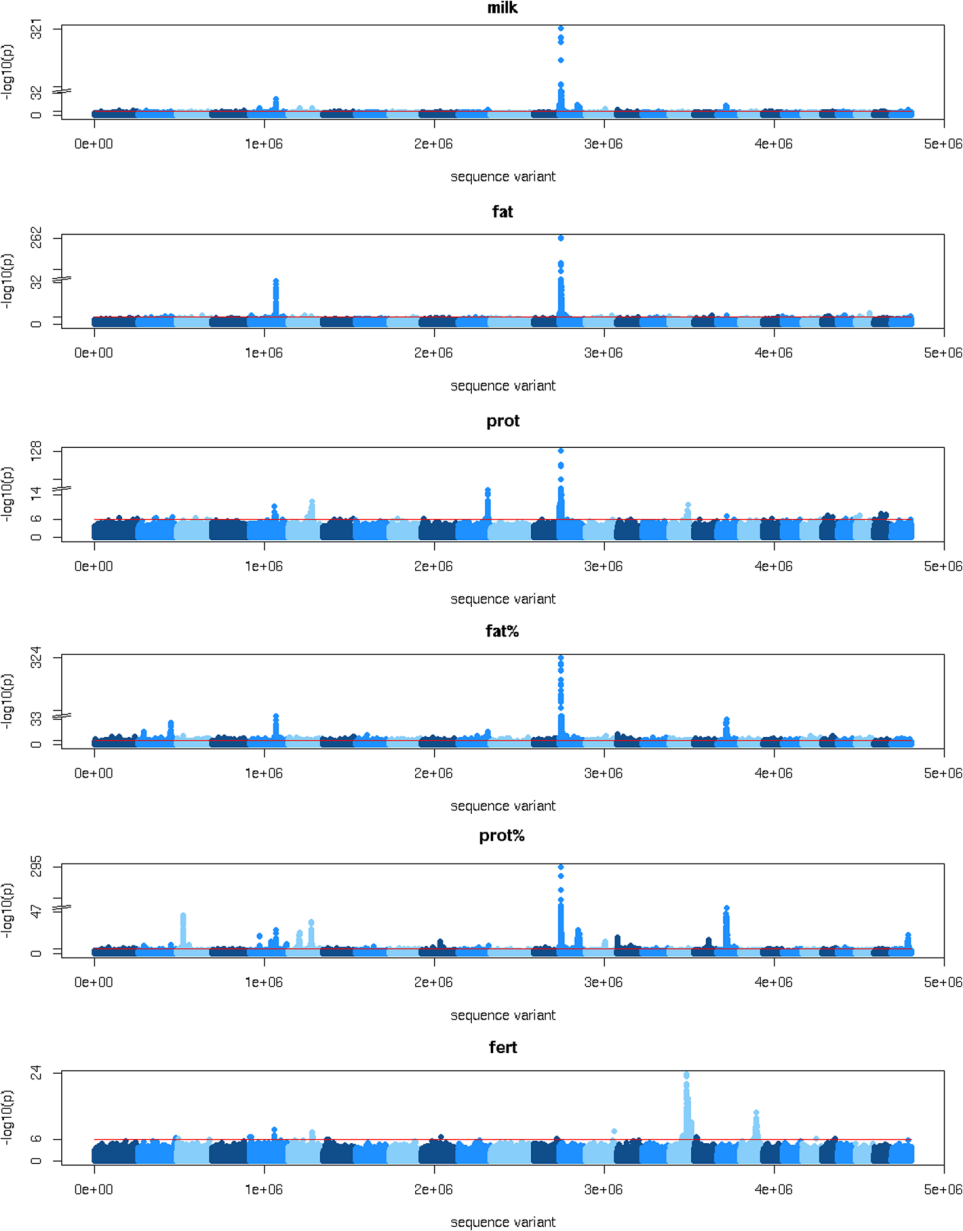
GWAS. Milk = milk yield, fat = fat yield, prot = protein yield, fat% = fat percentage, prot% = protein percentage, fert = fertility. The red line corresponds with a threshold of *p* ≤ 10^− 6^

**Fig. 2:**
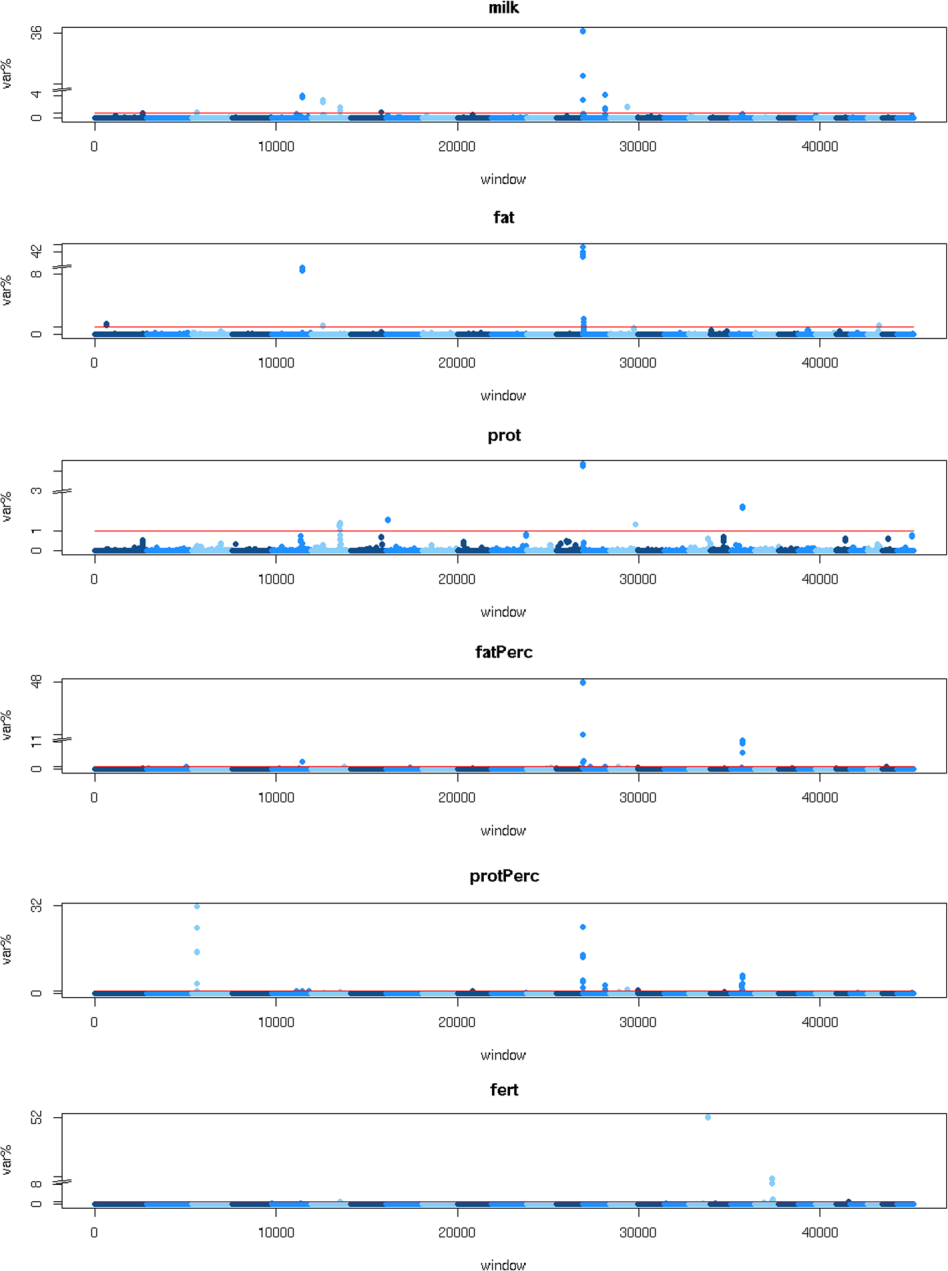
Local GEBV variance. Milk = milk yield, fat = fat yield, prot = protein yield, fat% = fat percentage, prot% = protein percentage, fert = fertility, var.% = variance explained by an interval as percentage of the sum of the variance explained by the non-overlapping intervals that explained the most variance. The red line corresponds with var.% = 1

We performed a GWAS using imputed sequence data and daughter trait deviations (DTD) and trait deviations (TD) for milk yield (milk), fat yield (fat), protein yield (prot), fat percentage (fat%), protein percentage (prot%) and fertility (fert) of up to 35,775 bulls and cows. The results of the GWAS are shown in Table 1 and Fig. 1. The number of GWAS variants with *p* ≤ 10^− 6^ ranged from 1204 for fert to 7017 for fat%, with a false discovery rate (FDR) of 0.007 and 0.001, respectively. Because a large number of sequence variants can be associated with the same QTL, we restricted the number of selected variants with p ≤ 10^− 6^ so that there was at least 1 Mb between variants. This reduced the number of variants, ranging from 89 variants selected for fertility to 861 for fat%. All chromosomes contained variants associated with production traits, with most variants located on chromosome 14, followed by chromosomes 20, 5, 6 and 3. Chromosomes 2, 3, 5, 6, 10, 13, 15, 18, 19, 21, 24 and 25 contained variants associated with fertility, with most variants located on chromosome 18, followed by chromosomes 21 and 6. We used the variant effects estimated by Bayes R hybrid to compute local GEBVs of 250 kb windows along the genome. The variance of these local GEBVs was then used to detect QTL. As shown in Fig. 2, the largest QTL detected by the local GEBV variances were located at the same locations as those detected using the GWAS.

**Table 1:** Number of significant GWAS variants and FDR per trait

trait	n	FDR	nQTL
milk	4333	0.001	213
fat	2136	0.003	140
prot	2287	0.003	165
fat%	7017	0.001	861
prot%	5298	0.001	307
fert	1204	0.007	89

*FDR* false discovery rate, *n* number of variants with a *p*-value below 10^− 6^, *nQTL* number of variants with a *p*-value below 10^− 6^, with at least 1 Mb between variants, *milk* milk yield, *fat* fat yield, *prot* protein yield, *fat%* fat percentage, *prot%* protein percentage, *fert* fertility

**Fig. 3:**
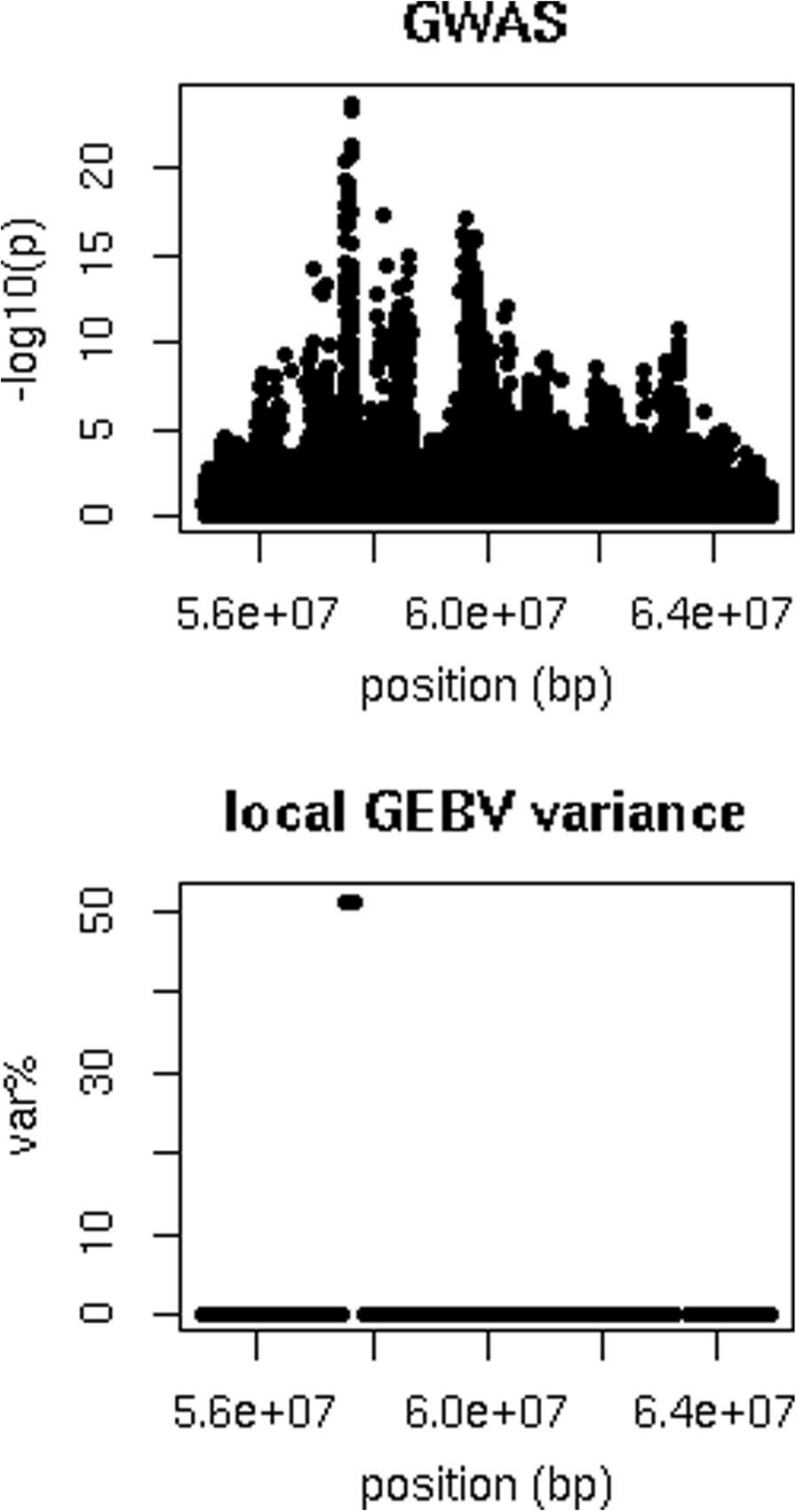
Comparison GWAS and local GEBV variance for a QTL on chromosome 18 for fertility. Position was the position of the sequence variant in the GWAS, or the middle of the 250 kb interval for which the local GEBV were calculated, var.% = variance explained by an interval as percentage of the sum of the variance explained by the non-overlapping intervals that explained the most variance

Figure 3 compares the precision of QTL mapping using either a GWAS or the local GEBV variance for a QTL on chromosome 18 detected for fert. The most significant variants in the GWAS were in an intron (*p* = 1.7 × 10^− 24^) and a missense variant (*p* = 5.3 × 10^− 24^) in *ENSBTAG00000037537*. The intervals that explained the largest part of $\sum {\sigma}_{locGEBV}^2$ were four overlapping intervals located between 57,565,406 and 57,696,310 bp, that explained 51% of $\sum {\sigma}_{locGEBV}^2$. In the GWAS many SNPs in this region are associated with the trait but it is unclear from the GWAS results whether this represents one or more QTL for fertility. However, the local GEBV variance shows only one peak, suggesting that there may be only one large QTL for fertility in this region.

### eQTL detection

RNA sequencing was used to obtain gene expression data of 105 Holstein and 26 Jersey cows. Milk samples were available for both Holstein and Jersey cows, blood samples only for Holstein cows. Subsequently, the association between 10,904,750 sequence variants and gene expression was estimated using a linear model. With a threshold of *p* ≤ 10^− 5^, there were 15,299 and 98,340 variants in the sequence data associated with the expression of 361 and 554 genes located within 1 Mb of the gene, using milk and white blood cells, respectively. The FDR corresponding with a threshold *p* ≤ 10^− 5^ was 0.06 and 0.01 for milk and white blood cells, respectively. All chromosomes contained eQTL.

### Correlations between local GEBV and gene expression

**Fig. 4:**
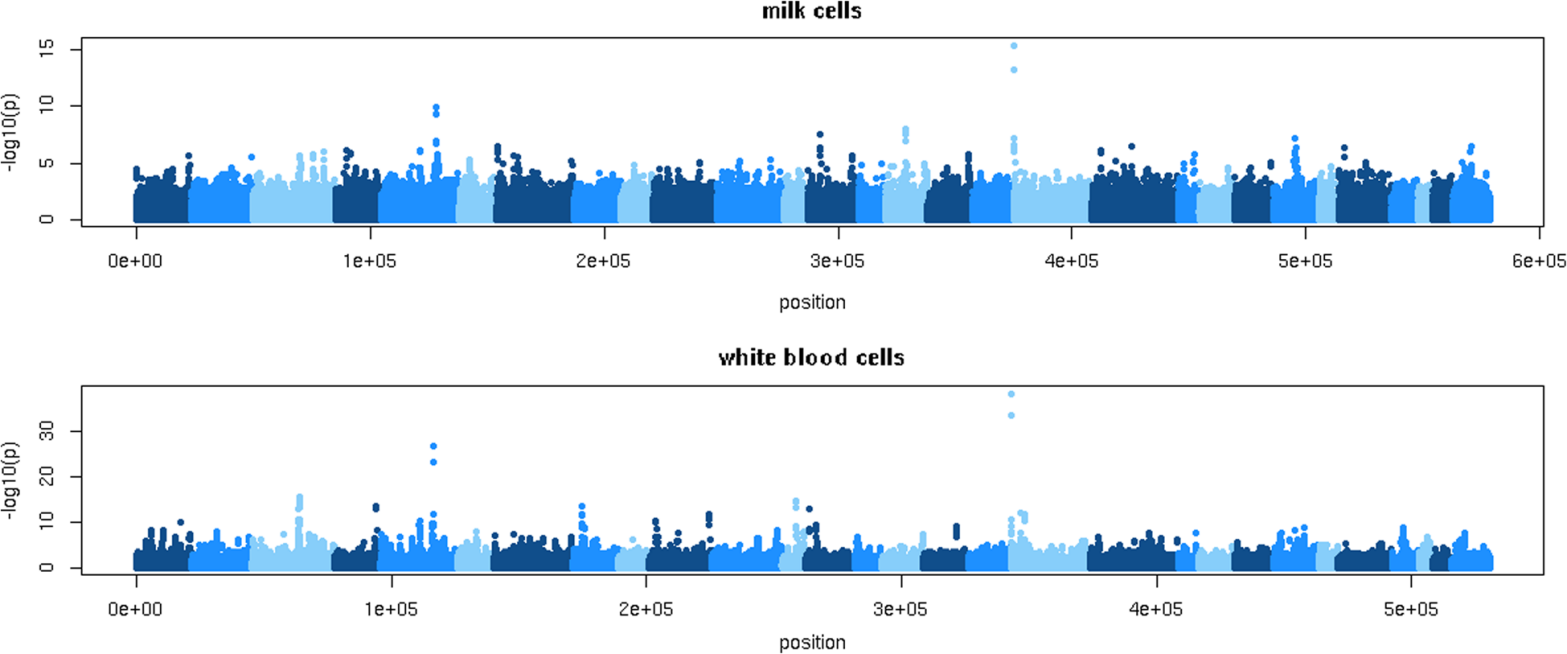
Correlations between local GEBV for fat yield and gene expression. Top = milk cells, bottom = white blood cells, correlations were estimated between 250 kb intervals and all genes within 1 Mb of the intervals, y-axis = −log10(*p*-value of the correlation between local GEBV and gene expression)

Correlations between local GEBV and gene expression was used to identify regions associated with both gene expression and the traits. Figure 4 shows the correlation between local GEBV for fat and the expression of genes within 1 Mb of the intervals for which the GEBV was calculated. Similar figures for the other traits can be found in Additional file 1. Significant correlations (p_cor(locGEBV,expr)_ ≤ 10^− 5^) were detected on all chromosomes, except for chromosomes 12, 24 and 27 using milk cells, and chromosome 28 using white blood cells. The most significant correlations were found on chromosome 18, followed by chromosome 5. Table 2 gives an overview of the number of correlations selected based on the p_cor(locGEBV,expr)_. In total, there were 3143 significant correlations. The majority of selected correlations, 2623, were detected using white blood cells, while only 520 correlations were detected using milk cells. The FDR ranged from 0.06 for milk, fat and prot% to 0.08 for fat% and fert using milk cells, and was 0.01 for all traits using blood cells. The variance in fat explained by the selected intervals was only a very small proportion of the total genetic variance. Using milk cells, the variance explained by selected intervals ranged from 0.03% for fert, to 0.43% for prot%. Intervals selected using white blood cells explained 3 to 19 times more variance then intervals selected using expression in milk cells. The largest percentage of variance was explained for prot%, where 147 selected intervals explained 2.57% of the total genetic variance.

**Table 2:** Overview of selected intervals based on the p-value of the correlation between local GEBV and gene expression, and the variance explained by the interval

cell type	trait	nSel	FDR	nInt	nGenes	%var
milk	milk	92	0.06	38	33	0.10
	fat	94	0.06	42	37	0.13
	prot	100	0.06	39	34	0.17
	fat%	70	0.08	28	27	0.09
	prot%	92	0.06	33	32	0.44
	fert	72	0.08	29	27	0.03
blood	milk	420	0.01	126	108	0.36
	fat	466	0.01	162	121	2.54
	prot	466	0.01	139	114	1.06
	fat%	414	0.01	136	118	0.62
	prot%	466	0.01	147	120	2.57
	fert	391	0.01	135	116	0.11

nSel = number of correlations between local GEBV and gene expression with a *p*-value ≤10^− 5^, *FDR* false discovery rate, *nInt* number of selected unique non-overlapping intervals, *nGenes* number of unique genes selected, *%var.* variance explained by the selected unique non-overlapping intervals as percentage of the sum of the variance explained by the non-overlapping intervals that explained the most variance, *milk* milk yield, *fat* fat yield, *prot* protein yield, *fat%* fat percentage, prot% protein percentage, *fert* fertility

**Fig. 5:**
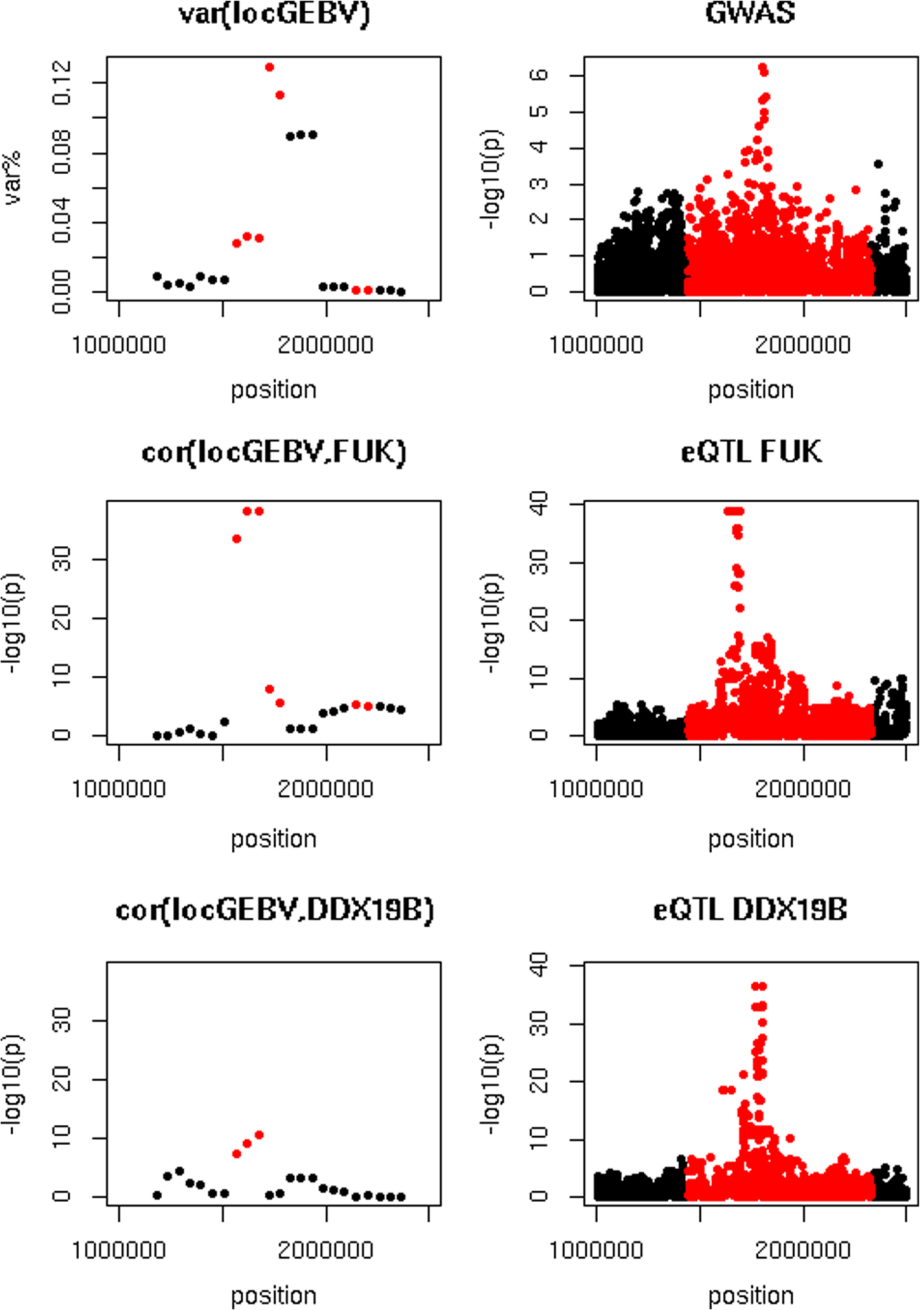
Association between *FUK* and *DDX19B* expression and fat yield. Top left = variance of local GEBV of 250 kb intervals where var.% = variance explained by an interval as percentage of the sum of the variance explained by the non-overlapping intervals that explained the most variance, top right = GWAS for fat yield, middle left = −log10(p) of correlations between local GEBV and *FUK* expression, middle right = association between sequence variants and *FUK* expression, bottom left = −log10(p) of correlations between local GEBV and *DDX19B* expression, bottom right = association between sequence variants and *DDX19B* expression. In all graphs, intervals or variants located within intervals with a p_cor(locGEBV,expr)_ ≤ 10^− 5^ are indicated in red

The most significant correlations were found on chromosome 18, where intervals located between 1,443,612 and 2,679,976 bp significantly correlated local GEBV for all traits with the expression of the gene fucokinase (*FUK),* and intervals located between 1,161,248 and 2,165,190 bp correlated local GEBV for all traits with the expression of the gene *DDX19B*. The strongest correlation equalled − 0.93 (*p* = 1.5 × 10^− 45^) and was detected between the local GEBV for fert for interval located from 1,703,787 to 1,953,787 bp with the expression of *DDX19B*. While there were highly significant correlations and eQTL in this region, the GWAS showed no association of any of the variants in the region with fertility and the local GEBV intervals explained at most 0.002% of $\sum {\sigma}_{locGEBV}^2$, as shown in Additional file 2. While all traits showed strong correlations between local GEBV and *FUK* and *DDX19B* expression, the GWAS only showed a peak for fat. Figure 5 compares the local GEBV variances, GWAS, correlation of local GEBV and *FUK* and *DDX19B* expression and eQTL study in this region, for fat. All analyses show a peak in the region, though not all at the same place.

The most significant GWAS hit for fat in the region was an intron variant in the *DDX19B* gene, with a *p*-value of 5.6 × 10^− 7^. The most significant correlation (*p* = 5.4 × 10^− 39^) with the gene expression of *FUK* was found with the local fat GEBV of the interval between 1,496,152 and 1,746,152 bp, with a correlation of 0.90. This interval contains part of the *FUK* gene, that is located from 1,681,355 to 1,694,462 bp, and explains 0.03% of $\sum {\sigma}_{locGEBV}^2$. There were 148 variants, located between 1,634,115 and 1,690,385 bp, in complete LD with each other in the individuals used for the eQTL study, that showed the strongest association with *FUK* expression in the eQTL study, with *p*-value of 1.1 × 10^− 39^.

The interval between 1,550,757 and 1,800,757 bp showed the strongest correlation between local fat GEBV and *DDX19B* expression, with a correlation of 0.59 and a p-value of 3.5 × 10^− 11^. This interval explained 0.03% of $\sum {\sigma}_{locGEBV}^2$, and has some overlap the *DDX19B* gene, that is located from 1,799,804 to 1,821,405 bp. The strongest association between sequence variants and *DDX19B* expression was detected for 17 variants, located from 1,802,850 to 1,804,766 bp with a *p*-value of 3.8 × 10^− 37^. Similar to the most significant eQTL for *FUK*, these variants were in complete LD in the individuals used for the eQTL study. While the most significant variants associated with *FUK* expression are not the most significant variants associated with *DDX19B* expression and vice versa, there were 375 variants located between 1 and 2.5 Mb with a p-value ≤10^− 5^ for both *FUK* and *DDX19B* expression.

The colocalization analysis did not find any evidence of colocalization for the GWAS QTL detected for fat and the eQTL for *FUK* and *DDX19B* expression. The largest CLPP were 7.3 × 10^− 5^ for *FUK* and 6.6 × 10^− 5^ for *DDX19B*. The genotypes of 148 top variants associated with *FUK* expression in the eQTL study did, however, all show a very strong correlation of − 0.98 (*p* = 6.2 × 10^− 95^) with the local GEBV between 1,496,152 and 1,746,152 bp. The top 17 eQTL variants associated with *DDX19B* expression had a correlation of − 0.35 (*p* = 3.6 × 10^− 5^) with the GEBV between 1,550,757 and 1,800,757 bp. For both genes, the direction of correlations and effects was consistent: the most significant SNPs in the eQTL study decreased both the expression of *FUK* and *DDX19B*, and the local GEBV, which was consistent with the positive genetic correlations between local GEBV and gene expression in the intervals.

Although the correlations between local GEBV and expression of genes are highly significant, we were concerned that they could arise by chance if SNPs that have a large contribution to the local EBV were also associated with expression of a nearby gene. This situation might arise due to widespread LD between SNPs within a segment. To test this possibility, we permuted the SNP effects within the local GEBV intervals and recalculated the correlation between these permuted local GEBV and gene expression. The number of correlations with *p* ≤ 10^− 5^, was only a little less than observed with the original GEBV (Table 3). The largest difference was found for fat and prot, where there were 466 intervals with significant correlations using the estimated variant effects and 345 intervals when the SNP effects within a local GEBV were permuted.

**Table 3:** Number of detected correlations using correct or permuted local GEBV

	milk	fat	prot	fat%	prot%	fert
correct	420	466	466	414	466	391
permuted	348	345	345	397	392	338

Correlations between gene expression and local GEBV with a *p*-value ≤10^−5^, variant effects used to estimate the genotypes were either the estimated effects or permuted within an interval, *milk* milk yield, *fat* fat yield, *prot* protein yield, *fat%* fat percentage, *prot%* protein percentage, *fert* fertility

### Overlap between top300 intervals and eQTL

The intervals with the highest correlations between local GEBV and gene expression included many intervals with very small effects on the milk production traits and fertility. This may have reduced our power to find QTL that are also eQTL. Therefore, we selected the 300 intervals that explained the most genetic variance for each trait (top300) and compared the number of eQTL they contained with the number of eQTL present in with 300 randomly selected intervals (random300). These intervals were spread over all chromosomes. Table 4 shows $\sum {\sigma}_{locGEBV}^2$ for the top300 and random300 intervals. The top300 intervals explained the majority of $\sum {\sigma}_{locGEBV}^2$, ranging from 74% for prot to 99% for fat% and prot%. A much smaller proportion of $\sum {\sigma}_{locGEBV}^2$ was explained by the random300 intervals. For all traits, the random300 intervals explained between 1 and 3% of $\sum {\sigma}_{locGEBV}^2$.

**Table 4:** Variance explained by top300 intervals and random300 intervals

intervals	milk	fat	prot	fat%	prot%	fert
top300	85%	82%	74%	99%	99%	93%
random300	3%	3%	3%	3%	2%	1%

Top300 were the 300 non-overlapping 250 kb windows that explained the most variance for each trait, random300 were randomly selected non-overlapping 250 kb windows, *chr* chromosome, *milk* milk yield, *fat* fat yield, *prot* protein yield, *fat%* fat percentage, *prot%* protein percentage, *fert* fertility, variance shows as percentage of the sum of the variance explained by the non-overlapping intervals that explained the most variance

Table 5 shows the number of top300 and random300 intervals containing eQTL, containing variants correlated with the local GEBV of the interval and having a significant correlation between the local GEBV of the interval and gene expression. Averaged across traits, 26 and 54 of the top300 intervals contained at least one eQTL using milk and white blood cells, respectively. Randomly selected intervals contained, on average across traits and replicates, 16 significant eQTL from milk and 36 from white blood cells. Thus, intervals with QTL were only slightly more likely to contain an eQTL than random intervals. Of the top300 intervals containing an eQTL, 7 and 28 also contained at least one variant whose genotype was both associated with gene expression and correlated with the local GEBV, averaged across traits using milk and white blood cells, respectively. Out of the random300 intervals, this was the case for 6 and 20 intervals, using milk and white blood cells, respectively.

**Table 5:** Number of top300 and random300 intervals containing eQTL, containing variants associated with local GEBV and showing a significant correlation between local GEBV and gene expression

		top300					random300				
cell type	trait	nE	nC	nEG	nEC	nEGC	nE	nC	nEG	nEC	nEGC
milk	milk	27	10	3	2	1	16	9	6	1	1
	fat	26	11	10	3	3	16	9	6	1	1
	prot	23	6	5	1	0	16	9	6	1	1
	fat%	25	11	9	1	1	16	8	5	1	0
	prot%	29	14	11	2	2	16	9	5	1	1
	fert	24	16	3	2	2	16	8	6	1	1
WBC	milk	58	20	27	13	11	36	15	20	6	5
	fat	48	18	28	12	10	36	15	20	6	6
	prot	45	24	23	10	9	36	14	20	6	5
	fat%	56	25	25	12	7	36	14	18	5	4
	prot%	61	25	39	10	10	36	14	19	6	5
	fert	55	30	24	12	8	36	16	21	6	6

Intervals were selected as the 300 non-overlapping intervals that explained most genetic variance (top300) or 300 randomly selected intervals (random300), the black dots show how many of the intervals containing at least one eQTL (nE) contain a variant that is associated with the eQTL and whose genotype correlates with the local GEBV of the interval (nEG), the blue dots show how many of the intervals with a significant correlation between local GEBV and gene expression (nC) contain an eQTL (nEC) that is correlated with the local GEBV of the interval, with consistent directions of effects (nEGC), *WBC* white blood cells, *milk* milk yield, *fat* fat yield, *prot* protein yield, *fatPerc* fat percentage, *protPerc* protein percentage, *fert* fertility

Only very few of the intervals showed a significant correlation between local GEBV and gene expression. Out of the top300 intervals, the number of intervals whose local GEBV correlated (*p* ≤ 10^− 3^) with the expression of a gene within 1 Mb of the interval ranged from 6 for protein to 16 for fertility using milk cells, and from 18 for fat to 30 for fertility using white blood cells. The majority of the top300 intervals, that showed a significant correlation between local GEBV and gene expression, did not contain a variant associated with both gene expression and the local GEBV. The number of top300 intervals fulfilling all criteria, with a significant correlation between local GEBV and gene expression, and containing a variant associated with both gene expression and the local GEBV equalled, averaged across traits, 2 for milk cells and 9 for white blood cells. Slightly fewer of the random300 intervals correlated with gene expression than the top300 intervals. Averaged across traits, 8 and 15 randomly selected intervals showed a significant correlation with gene expression, using milk and white blood cells, respectively, One and 5 of these contained at least one variant whose genotype was associated with gene expression and correlated with the local GEBV with effects consistent with the direction of the correlation.

Table 6 shows the top300 intervals whose local GEBV correlated with the expression of a gene within 1 Mb of the interval (*p* ≤ 10^− 5^) and that contained at least one variant associated with the expression of the gene (p ≤ 10^− 5^) that also correlated with the local GEBV of the interval (*p* ≤ 10^− 5^), with consistent effects and correlations (top300_EGC). Using milk cells, there were only three top300_EGC intervals, of which two of them were located on chromosome 18 and associated with the expression of *FUK* and GEBV for fat and prot%, and the third one located on chromosome 29 associated with *ENSBTAG00000037645* expression and milk GEBV. Using white blood cells, there were 25 top300_EGC intervals, of which 22 were associated with production traits and 3 with fertility. The top300_EGC intervals associated with production traits were located on chromosomes 3, 5, 11, 14, 18, 23 and 26, and the top300_EGC intervals associated with fertility were located on chromosomes 6, 10 and 21. The most significant eQTL in the top300_EGC intervals was an intron in *COG4* on chromosome 18 that was associated with the expression of *FUK* (*p* = 1.1 × 10^− 39^ and *p* = 13.8 × 10^− 11^ using white blood and milk cells, respectively) and correlated with the local GEBV for prot% (*p* = 7.0 × 10^− 38^ and *p* = 8.1 × 10^− 15^ using white blood and milk cells, respectively). The second most significant eQTL (*p* = 4.1 × 10^− 20^) was a variant downstream of mitochondrial ribosomal protein L51 (*MRPL51*) on chromosome 5 that was associated with the expression of non-SMC condensin I complex subunit D2 (*NCAPD2*) with a *p*-value of 4.1 × 10^− 20^ and correlated with the local GEBV for milk yield.

**Table 6:** Intervals containing eQTL and QTL

cell type	trait	chr	start (bp)	end (bp)	eQTL gene	topVar	annotation	p_eQTL	p_cor
milk	milk	29	42,610,883	42,860,883	*ENSBTAG00000037645*	42,829,338	5′ UTR *MARK2*	5.6 × 10^−06^	6.3 × 10^− 06^
milk	fat	18	1,601,923	1,851,923	*FUK*	1,840,070	intron *AARS*	5.1 × 10^− 06^	2.5 × 10^−07^
milk	prot%	18	1,550,757	1,800,757	*FUK*	1,666,278	intron *COG4*	3.8 × 10^−11^	8.1 × 10^−15^
WBC	milk	3	25,131,248	25,381,248	*SPAG17*	25,150,562	intron *SPAG17*	1.2 × 10^−06^	5.5 × 10^−12^
WBC	milk	5	104,173,293	104,423,293	*NCAPD2*	104,278,115	dns *MRPL51*	4.1 × 10^−20^	4.0 × 10^−07^
WBC	milk	18	18,190,766	18,440,766	*BRD7*	18,301,292	intron *ZNF423*	8.9 × 10^−06^	2.4 × 10^−10^
WBC	milk	23	31,068,838	31,318,838	*BTN1A1*	31,095,692	intergenic	6.3 × 10^−08^	1.5 × 10^−06^
WBC	milk	26	21,889,732	22,139,732	*TWNK*	21,901,063	ups *LBX1*	9.7 × 10^−07^	3.3 × 10^−07^
WBC	fat	14	2,753,849	3,003,849	*FAM83H*	2,755,467	synon LY6K	2.1 × 10^−06^	9.7 × 10^−06^
WBC	fat	18	1,601,923	1,851,923	*FUK*	1,612,928	ups *IL34* & dns *SF3B3*	2.6 × 10^−07^	8.6 × 10^−09^
WBC	fat	18	18,190,766	18,440,766	*BRD7*	18,301,292	intron *ZNF423*	8.9 × 10^−06^	3.1 × 10^−12^
WBC	fat	23	9,700,270	9,950,270	*C6orf222*	9,728,266	ups *CLPS*	1.3 × 10^−07^	2.9 × 10^−06^
WBC	fat	26	21,139,834	21,389,834	*TWNK*	21,142,651	intron *SCD*	5.4 × 10^−06^	5.3 × 10^− 06^
WBC	fat	26	21,889,732	22,139,732	*TWNK*	21,893,176	dns *LBX1*	1.5 × 10^−07^	2.6 × 10^−06^
WBC	prot	11	104,113,835	104,363,835	*ABO*	104,225,654	intergenic	3.8 × 10^−07^	5.9 × 10^−06^
WBC	prot	18	18,190,766	18,440,766	*BRD7*	18,301,292	intron *ZNF423*	8.9 × 10^−06^	9.3 × 10^−11^
WBC	fat%	5	75,536,412	75,786,412	*ENSBTAG00000012192*	75,596,191	intergenic	9.6 × 10^−07^	4.5 × 10^−06^
WBC	fat%	5	104,120,905	104,370,905	*NCAPD2*	104,139,091	intron *ZNF384*	3.6 × 10^−07^	2.2 × 10^−06^
WBC	fat%	26	22,005,004	22,255,004	*TWNK*	22,005,517	intron *BTRC*	5.4 × 10^−07^	1.9 × 10^− 07^
WBC	prot%	5	24,074,711	24,324,711	*CEP83*	24,074,711	intron *PLXNC1*	3.2 × 10^−06^	1.8 × 10^−12^
WBC	prot%	11	104,168,845	104,418,845	*ABO*	104,225,654	intergenic	3.8 × 10^−07^	4.2 × 10^−06^
WBC	prot%	14	2,132,257	2,382,257	*MAPK15*	2,236,999	splice *MAPK15*	4.7 × 10^−06^	2.2 × 10^− 06^
WBC	prot%	18	1,550,757	1,800,757	*FUK*	1,673,395	intron *COG4*	1.1 × 10^−39^	7.0 × 10^−38^
WBC	prot%	18	2,187,925	2,437,925	*FUK*	2,200,039	intron *FA2H*	3.4 × 10^−06^	3.6 × 10^−07^
WBC	prot%	26	24,899,228	25,149,228	*ENSBTAG00000038540*	25,027,259	intron *CFAP43*	1.3 × 10^−06^	2.3 × 10^−08^
WBC	fert	6	38,538,611	38,788,611	*FAM184B*	38,585,743	intron *LAP3*	9.5 × 10^−07^	9.8 × 10^−06^
WBC	fert	10	37,985,065	38,235,065	*CDAN1*	38,017,802	intergenic	3.0 × 10^−07^	2.0 × 10^−08^
WBC	fert	21	48,591,579	48,841,579	*SSTR1*	48,641,167	intergenic	6.9 × 10^−06^	1.8 × 10^−06^

*Chr* chromosome, *topVar* position in basepair (pb) of variant with strongest association with eQTL and localGEBV, *annotation* functional annotation of topVar, *dns* downstream, *ups* upstream, *p_eQTL p*-value eQTL analysis, *p_cor p*-value correlation local GEBV and gene expression, *WBC* white blood cells

Genes whose expression correlated with the local GEBV of top300_EGC for multiple production traits were *NCAPD2* on chromosome 5 associated with milk and fat%, alpha 1–3-N-acetylgalactosaminyltransferase and alpha 1–3-galactosyltransferase (*ABO*) on chromosome 11 associated with prot and prot%, *FUK* on chromosome 18 associated with fat and prot% (*BRD7*) on chromosome 18 associated with milk, fat and prot, and twinkle mtDNA helicase (*TWNK*) on chromosome 26 associated with milk, fat and fat%.

### Colocalisation of QTL and eQTL

Using the programme eCAVIAR, we examined the intervals with significant QTL to see if there was a SNP with a high posterior probability that was both the QTL and an eQTL by estimating the probability that a variant is causal in both the GWAS and eQTL analysis, the colocalization posterior probability (CLPP). Variants were considered to colocalise if they had a CLPP ≥0.01. The majority of QTL selected from the GWAS did not colocalise with eQTL (Table 7). For instance, out of 64 milk QTL, only 4 had a single SNP with a posterior probability (CLPP) > 0.01 that it is both the QTL and the eQTL. More variants colocalised when white blood cells were used then when milk cells were used. Averaged across traits, 3.7 and 6.8% of the GWAS QTL colocalised with an eQTL for milk and white blood cells, respectively. When we started with intervals that contained an eQTL, only between 1 and 3% of the eQTL colocalised with QTL for the traits in our analysis, using either milk or white blood cells.

**Table 7:** Colocalisation between QTL and eQTL

cell type	trait	n_QTL	coloc_QTL	n_eQTL	coloc_eQTL
milk	milk	64	4	361	4
	fat	39	3	361	6
	prot	45	2	361	4
	fat%	9773	34	361	4
	prot%	3455	13	361	3
	fert	30	1	361	4
WBC	milk	64	7	554	8
	fat	39	3	554	7
	prot	45	2	554	8
	fat%	9773	26	554	9
	prot%	3455	19	554	15
	fert	30	5	554	6

*WBC* white blood cell, n_QTL = the number of variants with a GWAS p-value ≤10^−5^, counting maximum 1 QTL per 1 Mb, coloc_QTL = the number of QTL with at least one variant with a colocalization posterior probability (CLPP) ≥ 0.01, n_eQTL = the number of genes with at least eQTL variant within 1 Mb of the gene with a *p*-value ≤10^− 5^, coloc_eQTL = the number of eQTL with at least one variant with a colocalization posterior probability (CLPP) ≥ 0.01

**Fig. 6:**
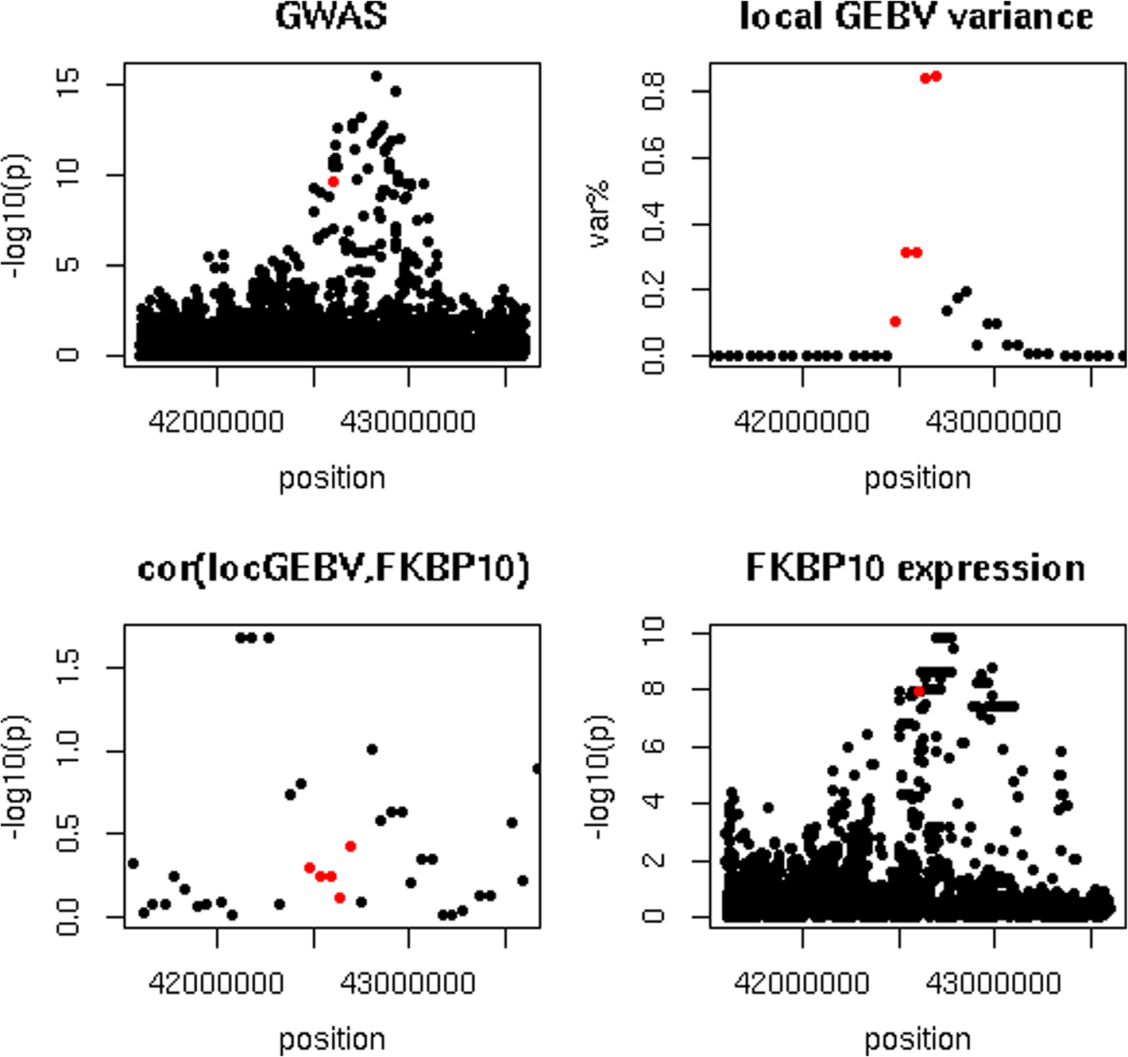
Protein percentage GWAS and eQTL study results for chromosome 19 around *FKBP10.* Top left = GWAS for protein percentage, top right = local GEBV variance where var.% = variance explained by an interval as percentage of the sum of the variance explained by the non-overlapping intervals that explained the most variance, bottom left = correlation between local GEBV and *FKBP10* expression, bottom right = eQTL study for *FKBP10* expression, red dot indicates variant or intervals containing variant with largest colocalisation posterior probability (CLPP)

Table 8 shows the top 10 variants with the highest CLPP, located on chromosomes 3, 5, 8, 18, 19 and 25. The highest CLPP was 1.00 and detected for a variant downstream of junction plakoglobin (*JUP*) around 43 Mb on chromosome 19 that was associated with the expression of FK506 binding protein 10 (*FKBP10*). This variant had a *p*-value of 2.4 × 10^− 10^ and 1.2 × 10^− 3^ in the GWAS for prot% and fat%, respectively, and a p-value of 1.2 × 10^− 8^ in the eQTL study. Figure 6 shows the GWAS, local GEBV variance, eQTL study and correlation between local GEBV and gene expression of the area around this variant. Local GEBV intervals containing the variant with the highest CLPP explained at most 0.8% of $\sum {\sigma}_{locGEBV}^2$ for prot% and fat%, respectively.

**Table 8:** Top 10 colocalizing variants

cell type	trait	chr	gene	position	annotation	CLPP
WBC	prot%	19	*FKBP10*	42,601,890	dnst *JUP*	1.00
WBC	fat%	19	*FKBP10*	42,601,890	dnst *JUP*	1.00
WBC	prot	18	*PRMT1*	57,135,434	intergenic	0.95
WBC	prot%	5	*DIP2B*	30,479,476	intron *SPATS2*	0.95
milk	milk	5	*PWP1*	70,897,603	intergenic	0.74
WBC	fat	18	*BRD7*	18,411,077	intergenic	0.74
WBC	prot	3	*ANKRD35*	21,083,019	intergenic	0.69
milk	milk	25	*HBQ1*	494,716	splice region variant *PRR35*	0.68
WBC	prot%	25	*TFR2*	36,092,765	dnst ENSBTAG00000020157	0.68
milk	fat%	8	*PTPDC1*	87,424,582	intron *ROR2*	0.59

*WBC* white blood cells, *CLPP* colocalization posterior probability, *dnst* downstream

### Validation of known QTL and eQTL

**Fig. 7:**
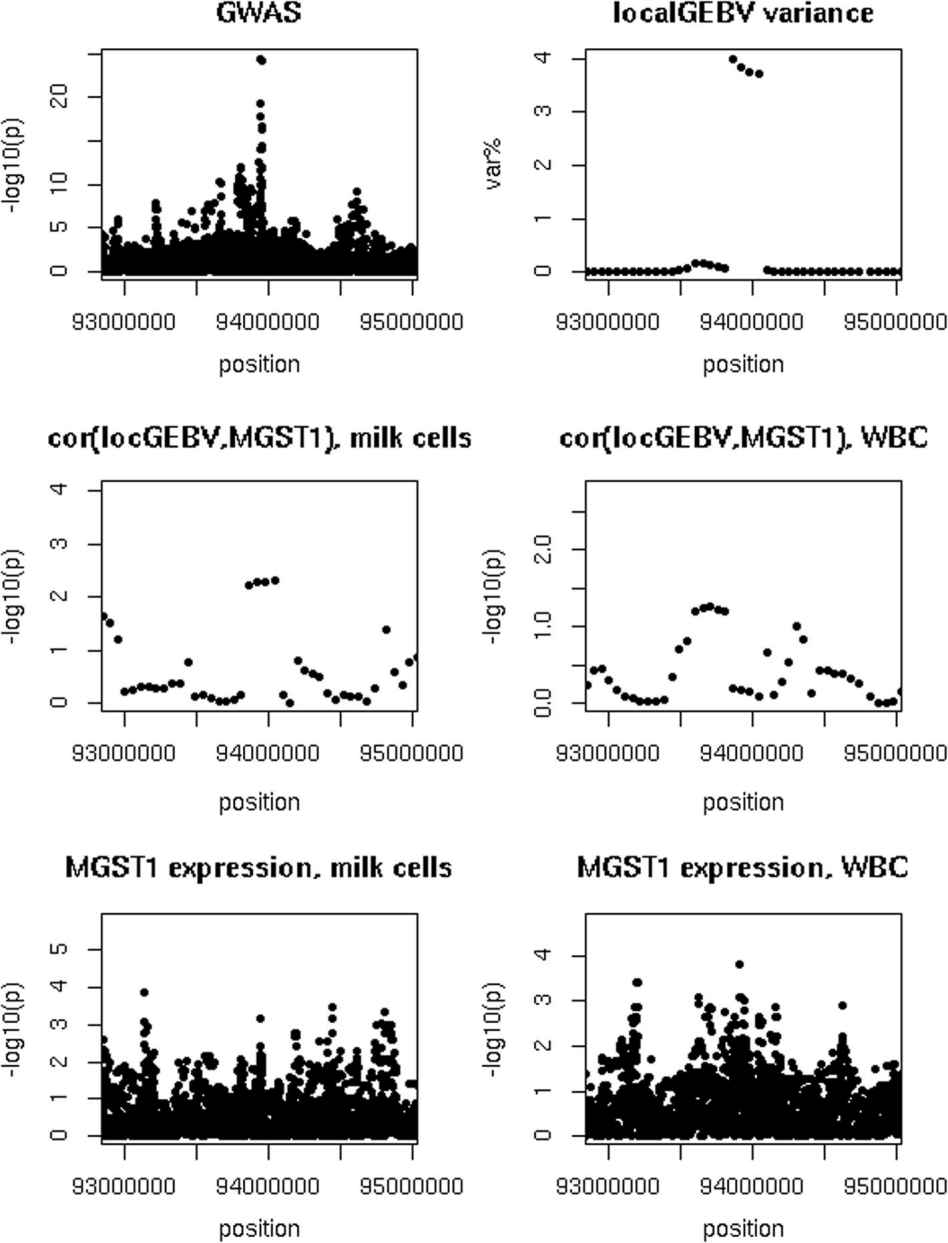
GWAS and eQTL study results for chromosome 5 around *MGST1.* Top left = GWAS for fat percentage, top right = variance of GEBV of 250 kb intervals where var.% = variance explained by an interval as percentage of the sum of the variance explained by the non-overlapping intervals that explained the most variance, middle left = *p*-value of correlation between local GEBV in 250 kb intervals and *MGST1* expression using milk cells, middle right = *p*-value of correlation between local GEBV in 250 kb intervals and *MGST1* expression using white blood cells, bottom left = eQTL study for *MGST1* expression using milk cells, bottom right = eQTL study for *MGST1* expression using white blood cells

As shown in Fig. 7, there was a clear peak in both the GWAS and the local GEBV variance for fat% around *MGST1*. The strongest correlations with *MGST1* expression were a correlation of 0.26 (*p* = 4.1 × 10^− 3^) found for an interval between 93,798,862 and 94,048,862 bp using milk cells, and of − 0.20 (*p* = 0.04) for an interval between 93,579,668 and 93,829,668 bp using white blood cells. The most significant variants associated with *MGST1* expression for milk and white blood cells were intergenic variants located at 93,133,977 (*p* = 1.3 × 10^− 4^) and 93,911,186 (*p* = 1.6 × 10^− 4^), respectively.

Additional file 3 shows the GWAS, local GEBV variance, correlation between local GEBV and gene expression and eQTL analysis for milk yield in the area around *SLC37A1* on chromosome 1. The most significant GWAS variant was an intergenic variant with a *p*-value of 2.5 × 10^− 8^. This variant was located in the interval between 144,244,143 and 144,494,143 bp that explained the largest percentage of $\sum {\sigma}_{locGEBV}^2$ of intervals in the region (1%). There are SNPs weakly associated with expression of *SLC37A1* in milk cells but not in white blood cells. Similar to *SLC37A1,* the QTL around the *PAEP* gene on chromosome 11 was detected using both the GWAS and the local GEBV variance (Additional file 4). Again, there is a SNP weakly associated with expression of *PAEP* in milk and weak correlations between local EBVs and *PAEP* expression in milk cells (results are only shown for milk cells and not for white blood cells, because in the eQTL analysis using white blood cells, *PAEP* was excluded because it was not expressed in sufficient animals.

## Discussion

We combined results from GWAS, local GEBV variances, an eQTL study and a colocalisation analysis to detect regions that were associated both with quantitative traits in dairy cattle and gene expression. We used three strategies to identify regions and variants that were associated both with gene expression and quantitative traits in dairy cattle: selecting intervals that showed a strong correlation between local GEBV and gene expression, looking for eQTL in regions that were among the 300 intervals that explained most of the genetic variants for a certain trait, and calculating the probability that a variant was causal for both an eQTL and a QTL. While we identified several regions associated both with QTL and eQTL, we did not find eQTL that explained the majority of QTL for milk production and fertility.

### QTL detection using GWAS and local GEBV variances

To detect variants and regions associated with production traits and fertility, we used both a GWAS and variances of local GEBV computed using variants effects estimated with Bayes R. With both approaches, multiple QTL were detected, mainly confirming well-known QTL. Using local GEBV variance resulted in a more precise signal, while with the GWAS, peaks covered a larger area of the genome. This is in line with other studies that have demonstrated an improved precision of QTL detection with Bayes R compared to GWAS and GBLUP [12, 13]. As a consequence of the long range LD present in dairy cattle [6], variants relatively far away from the causative mutation can still be in LD with the causative mutation, resulting in broad GWAS peaks. By estimating all variant effects simultaneously, Bayes R effect estimates can be more precise. If several variants are in almost complete LD, Bayes R will calculate low posterior probabilities for all of them. Therefore, we used the local GEBV variance, that combines the effects of variants in a region. Estimating variants effects for full sequence data using a Bayesian model is, however, computationally challenging [15, 16]. Therefore, we used the local GEBV intervals based on HD effects estimated with Bayes R to detect QTL regions, and used the GWAS for the colocalisation analysis and to zoom into sequence level.

None of the methods we used give an accurate estimate of the number of independent QTL. Because LD is conserved over long distances in dairy cattle [6], it is difficult to disentangle individual QTL, and therefore, the number of QTL reported here should not be interpreted as independent QTL.

### Correlations between local GEBV and gene expression

While some intervals showed a very strong correlation between local GEBV and gene expression, these intervals explained a minimal part of the total genetic variance, and would not be classified as QTL using a minimal threshold required to avoid a large number of false positives. Quantitative traits are influenced by many causative variants, and it is possible that individual causative variants explain only a very small part of the total genetic variance, in the same range as the variance explained by the intervals we detected. In the regions we detected, it was difficult to identify a sequence variant that showed a strong association with both gene expression and quantitative traits, and permutation analysis showed that most, but not all, of the significant correlations between local GEBV and gene expression could be due to chance, generated by the high local LD. That is, any linear combination of SNP genotypes (such as a local GEBV) will be correlated with many SNPs in the region and if one of these is associated with expression of a gene, it will generate a correlation between the local GEBV and gene expression.

The most significant correlations between local GEBV and gene expression were found between production traits and the expression of *FUK* and *DDX19B*. *FUK* has been reported by Ibeagha-Awemu et al. [4] as a candidate gene for milk traits because of its association with butyric acid levels (C4:0). *DDX19B* is involved in molecular transport in human [17] and has been associated with organismal, organ and tissue development in pigs [18]. While our results show evidence for eQTL for both *FUK* and *DDX19B* expression in the region, it is not clear whether there is one eQTL affecting both genes or two different eQTL. There were variants significantly associated with the expression of both genes, but the most significant variants detected for either gene were at different locations, suggesting that there may be two eQTL present in the region, that are in LD with each other.

### Overlap between top300 and random300 intervals and eQTL

Our second method of finding QTL that are also eQTL focused on chromosome intervals that contained larger QTL for milk traits or fertility. We selected the 300 intervals that explained the largest part of the total GEBV variance, and compared the number of eQTL present in those intervals to the number of eQTL in randomly selected intervals. While the top300 intervals explained a much larger proportion of the total GEBV variance than randomly selected intervals, there were only slightly more eQTL present in the top300 intervals than in randomly selected intervals. This reflects how common eQTL are. Our results suggest that if there is an eQTL near a QTL, it could be simply due to the abundance of eQTL. Therefore, we looked for evidence that the QTL and the eQTL were indeed identical by applying a series of filters to the intervals that were in the top300 and which contained an eQTL. Specifically, we looked for intervals that contained a SNP associated with both the gene expression and the local GEBV and in which the local GEBV was correlated with gene expression. Only few of the top 300 intervals fulfilled all criteria, suggesting that while there were eQTL present in most intervals, only few of them could be associated with the local GEBV of the intervals.

For most of the genes associated with the top300_EGC intervals, we could not find information in the literature linking their effects to the trait the interval was selected for. Exceptions to this were *ABO* on chromosome 11, and *BRD7* [19] and the previously described *FUK* on chromosome 18. On chromosome 11, a well-known QTL for production traits in dairy cattle is located near the beta-lactoglobulin precursor (*PAEP*) gene around 103.3 Mb [20]. While we did not find any eQTL associated with *PAEP*, an interval between 104.1 and 104.4 Mb contained an eQTL associated with the expression of *ABO*. The local GEBV for protein and prot% of the interval also correlated with *ABO* expression. *ABO* influences blood type in human [21]. Due to the proximity of *ABO* to *PAEP*, it is difficult to say whether there are two separate QTL segregating in the region or whether the peak near *ABO* is actually still due to the *PAEP* QTL. As shown in Additional file 4, while the major peak in both the GWAS and local GEBV variance is around *PAEP*, a second, smaller peak is visible near *ABO*, suggesting that there may be two QTL present in this area.

The top300_EGC intervals contained several intervals on chromosome 26 located between 21.9 and 25.1 Mb associated with milk, fat, fat% and prot%. The local GEBV in these intervals were either associated with the expression of twinkle mtDNA helicase (*TWNK*), Steroid 17-alpha-hydroxylase/17,20 lyase precursor (*CYP17A1*) or *ENSBTAG00000038540*. While there is no apparent link between any of these genes and milk production traits, QTL in this region have been associated with various traits in dairy cattle, including production traits [20, 22]. Other studies have suggested *SCD* as a candidate gene underlying this QTL [20, 23]. While there was an intronic SNP in *SCD* associated with *TWNK* expression, we did not find an eQTL associated with *SCD* expression, nor a significant correlation between any interval in the region and *SCD* expression. Another gene containing variants associated with *TWNK* expression was *BTRC*, that has a been associated with a large range of effects in mice and human (e.g. [24, 25]), including mammary gland development [24]. Similar to *SCD*, no eQTL or correlation associated with *BTRC* expression were detected.

### Colocalisation of eQTL and QTL

Thirdly, we used the CLPP to compute how many eQTL and QTL colocalised. We only found very few variants that colocalised, and none of them were located in major QTL. CLPP lacks power due to the high LD which meant that posterior probabilities were usually split among many SNPs in high LD with each other. Therefore, CLPP may not be a suitable method in livestock datasets where LD is conserved over long distances. The largest CLPP was found for an intergenic variant associated with *PRMT1* expression. *PRMT1* is essential for protein arginine methylation in mice [26].

### Known QTL and eQTL

There is a major QTL associated with fat yield near the *MGST1* gene, and Littlejohn et al. [10] detected an eQTL for *MGST1* expression using mammary tissues of 375 lactating cows. While there was a clear peak visible around the *MGST1* gene in both the GWAS and local GEBV for production traits, correlations between local GEBV and *MGST1* expression were weak, and only small peaks were visible in the eQTL study. Thus, we could be said to confirm result of Littlejohn et al. [10], but if we used such low significance thresholds genome wide, we would find too many false positives. An explanation for the weak evidence in our dataset could be the difference in size of the dataset and type of tissues used for the analysis. Littlejohn et al. [10] used mammary tissue samples from 375 lactating cows while our dataset contained white blood cell samples of 105 individuals and milk cell samples of 131 individuals. Similarly, in the case of *SLC37A1* and *PAEP*, we found only weak evidence that these QTL are actually eQTL.

### Limitations

It is possible that our datasets lack power to show that QTL are, in fact, eQTL. While our datasets are not small by agricultural standards (37,000 phenotypes and 131 cows with gene expression), larger numbers would increase power and mapping precision. Furthermore, it may be that we did not sample the right tissues at the correct age and physiological state. Though milk cell samples do mimic the gene expression of the lactating mammary gland, the variation in cell types and health did result in greater variation in gene expression and therefore less power to detect eQTL. This was evident by detecting less eQTL in milk cells than in white blood cells. Furthermore, we performed genome wide analyses rather than focussing on specific regions, so a stringent significance threshold was required to avoid false positives, reducing the power to detect QTL, eQTL and correlations between local GEBV and gene expression. The long-range LD in dairy cattle imposes further limitations, both in the detection of correlations between gene expression and local GEBV, and for the precision of QTL and eQTL detection. The combination of the small dataset, necessity of stringent thresholds to avoid false positives and the long-range LD may explain why we found little overlap between QTL and eQTL. For future eQTL studies, we would recommend a larger dataset, though it is difficult to predict what the minimum sample size should be. Based on the more promising results reported by Littlejohn et al. [10], we would suggest a dataset containing at least 300–400 individuals.

Besides the limitations of our study to identify QTL that are eQTL, it may be that eQTL explain only a small fraction of QTL. As well as differences in gene expression, QTL might affect amino acid sequence, splicing of transcripts, post-transcription and post-translational changes. For instance, Kemper et al. [11] found a QTL that affects casein concentration but not corresponding RNA concentration in mammary tissue. In studies of humans, the enrichment of eQTL among GWAS hits is not very high (e.g. 2 times). This implies that most QTL may not be eQTL or at least not eQTL discovered in the reported studies.

### Multi breed QTL detection

In our study, we used a multi breed population for all QTL detection methods. However, not all QTL segregate across breeds [12, 27]. While using a multi breed population can lead to improved QTL detection precision [12, 28], results can be dominated by the breed with the largest population size [28]. For QTL that segregate across breed, a multi breed population increases the sample size and therefore the power. Furthermore, because LD is conserved over shorter distances across breed than within breed [6], fewer variants and only variants closer to the causative mutation would be expected to show a significant association.

Breed specific QTL can, however, be overshadowed by a nearby QTL segregating in a breed with a substantially larger population size [28]. Consequently, by performing multi breed analysis rather than within breed analysis, we may have missed some breed specific QTL, especially QTL specific for Jersey. Because the focus of this paper was to study the overlap between QTL and eQTL, and the number of Jersey cows with expression data was very small our analysis would not be powerful enough to focus on breed specific QTL. Therefore, we preferred doing only a multi breed analysis, to benefit from the improved precision.

In future studies, with larger within breed populations, it will be interesting to compare breed specificity of eQTL and the overlap between breed specific QTL and breed specific eQTL.

## Methods

### Genotypes

Genotypes were available for 35,775 Holstein, Jersey and crossbred bulls and cows, the reference population is described in more detail by van den Berg et al. [16]. All of these were genotyped with the Illumina 10 K, Illumina BovineSNP50 (50 K) chip or the Illumina 800 K BovineHD (HD) bead chip. The animals genotyped at a lower density were first imputed up to HD genotypes, a total of 632,003 variants. Subsequently, all individuals were further imputed up to sequence level, using a reference population consisting of Holstein, Jersey and Australian Red bulls and cows from Run 5 of the 1000 bulls genome project [29]. All imputation was done using FImpute [30]. After removing variants with a minor allele frequency (MAF) below 0.002 and LD pruning (r^2^ > 0.9), the sequence dataset (SEQ) contained 4,812,745 variants. Because further analysis was performed using a multi breed population and we did not focus on breed specific QTL, we performed both MAF filtering and LD pruning across populations. The LD pruning was performed to reduce the number of variants, reducing the computational demand and by reducing the number of tests, reducing the number of false positives in the GWAS. The MAF filtering aimed to remove very rare variants that would likely have had a low imputation accuracy.

### Phenotypes

Daughter trait deviations (DTD) and trait deviations (TD) were used as phenotypes for bulls and cows, respectively. All 35,775 individuals had DTD or TD for milk yield (milk), fat yield (fat) and protein yield (prot). Fat percentage (fat%) and protein percentage (prot%) were available for 32,923 Holstein and Jersey bulls and cows, and fertility (fert), measured as calving interval in days, for 32,819 Holstein, Jersey and crossbreed bulls and cows.

### GWAS

The SEQ dataset and phenotypes for milk, fat, prot, fat%, prot% and fert were used in a GWAS to estimate the effects of sequence variants on each trait. First, GWAS were carried out using the mixed linear model analysis as implemented in GCTA [31], fitting the HD genomic relationship matrix to account for population structure and the genotype of a variant and breed as fixed effects. GWAS were done separately for bulls and cows, because of the higher accuracy of bulls DTD compared to cows TD, and a weighted analysis is currently not possible in GCTA. Within sex, differences in DTD and TD accuracies are much smaller, allowing us to do an unweighted analysis. The estimated *p*-values and directions of the effects in the within sex GWAS were combined in a meta-analysis, using the weighted z-score model as implemented in METAL [32].

### Estimation of local GEBV

To calculate local GEBV, we used Bayes R hybrid [33] to estimate effects for all the HD SNP for all traits. Estimating SNP effects was done using the 35,775 individuals with HD genotypes and DTD/TD. The SNP effects were then used to estimate local GEBV for the individuals in the gene expression dataset. Similar to Kemper et al. [12], we summed up SNP effects over sliding windows of 250 kb, to obtain local GEBV, with 50 kb between the start positions of adjacent windows. The variance of the local GEBV was used for QTL mapping, and compared to the sum of the variance explained by the non-overlapping intervals that explained the most variance ($\sum {\sigma}_{locGEBV}^2$). For this, all intervals were first ranked based on their variance, and subsequently, intervals overlapping with an interval that explained more variance were removed, until only non-overlapping intervals remained. $\sum {\sigma}_{locGEBV}^2$ was then calculated as $\sum {\sigma}_{locGEBV}^2=\sum \limits_{u=1}^U\mathit{\operatorname{var}}{(locGEBV)}_u$, where *U* was the number of non-overlapping intervals that explained the most variance.

To study the effect of LD in the local GEBV window on the estimated correlations, we permuted the variant effects within each window and used the permuted effects to estimate permuted local GEBV. This permutation test tests the null hypothesis that the correlation between local GEBV and the expression of a gene could occur by chance, due to high LD between variants in the local GEBV window. A variant that has a large contribution to the local GEBV may be in LD with a mutation that affects gene expression. The permutation test was repeated 100 times.

### Gene expression data and eQTL detection

Gene expression was obtained from RNA sequencing of milk and white blood cell samples. Milk samples were collected from 105 Holstein and 26 Jersey cows, and blood samples from 105 Holstein cows. A more detailed description of the pipeline to obtain the gene expression data can be found in [34]. HD and imputed sequence data were available for all these individuals. The sequence data for the eQTL study (ESEQ) was filtered based on MAF for the cows used in the analysis and no LD pruning was performed, resulting in 10,904,750 and 10,469,612 variants used for the milk and blood study, respectively. We used log transformed read counts as a measure of gene expression levels. Only genes expressed in at least 25 cows were analysed, which reduced the number of genes to 12,772 in milk cells and 11,577 in white blood cells. For each variant in the ESEQ dataset, the association with the expression of each gene within 1 Mb of the variant was estimated using a linear model in EMMAX [35], fitting the HD genomic relationship matrix to account for population structure and the genotype of the variant, breed, parity, days in milk and RNA sequencing batch as fixed effects. We only considered cis-eQTL in this study to reduce the number of tests. A larger number of tests would have required a more stringent detection threshold, reducing detection power. Furthermore, we expected cis-eQTL to be larger than trans eQTL, and therefore easier to detected.

### Correlating local GEBV with gene expression

To detect regions that were associated with both gene expression and the traits, we estimated Pearson’s correlations between the local GEBV and gene expression. We estimated correlations between local GEBV intervals and each gene within 1 Mb of an interval. Subsequently, we selected all intervals with a correlation that had a *p*-value ≤10^− 5^.

### Correlating genotypes with local GEBV

To determine which sequence SNPs were associated with the local GEBV of an interval, we computed the correlation between the genotypes of SEQ variants in an interval with the local GEBV of that interval.

### Comparing of top300 and random300 intervals

To test whether QTL regions were enriched for eQTL, we selected 300 non-overlapping intervals per trait that explained the most of the GEBV variance (top300) and compared those to 300 randomly selected intervals (random300). The selection of random300 intervals was repeated 100 times. We then determined how many of the top300 and random300 intervals contained a variant associated with the expression of a gene within 1 Mb of the interval with a p-value ≤10^− 5^ (nE), and a variant correlated with the local GEBV of the interval with a p-value ≤10^− 5^ (nG), if there was a variant both associated with the expression of a gene within 1 Mb of the interval with a p-value ≤10^− 5^ and correlated with the local GEBV of the variant with a *p*-value ≤10^− 5^ (nEG), and if the local GEBV were correlated with the expression of a gene within 1 Mb of the interval with a p-value ≤10^− 3^ (nC). For intervals fulfilling all criteria, we compared whether the direction of effects was consistent, hence if a variant had a positive effect on gene expression and was negatively correlated to the local GEBV of the interval, the correlation between the local GEBV in the interval should be negative (nEGC).

### Colocalisation of QTL and eQTL

To estimate the probability that the same variant is causal in both the GWAS and the eQTL analysis, we used eCaviar [14]. eCaviar computes the colocalisation posterior probability (CLPP) based on the marginal statistics for a GWAS (*S*^*(p)*^) and eQTL study (*S*^*(e)*^). *S*^*(p)*^ and *S*^*(e)*^ were computed based on the effects estimated in the GWAS and eQTL analysis, respectively, and the LD between variants. Following Hormozdiari et al. [14], variants with a CLPP ≥0.01 were considered to be shared between the GWAS and eQTL study.

To estimate how many QTL and eQTL were colocalising, we first selected QTL and eQTL based on the *p*-values estimated in the GWAS and expression analysis. A QTL was defined as a region that contained at least one GWAS variant with a p-value ≤10^− 6^, with at least 1 Mb between adjacent QTL. We first selected all genes that were located within 1 Mb of the QTL. Subsequently, we computed the CLPP for all sequence variants located within 1 Mb of these genes. An eQTL was defined as a variant with a p-value ≤10^− 5^ in the eQTL analysis, with maximum 1 eQTL per gene. For each eQTL we computed the CLPP for all variants within 1 Mb of the gene.

### Validation of known QTL and eQTL

*MGST1* is a well-known QTL that has been detected as an eQTL in dairy cattle [10], and *SLC37A1* and *PAEP* have been reported to be differentially expressed in lactating mammary tissue than in other tissues [20]. We used the results of all above described methods in an attempt to validate our methods and the presence of these QTL and eQTL in our dataset.

### False discovery rate

For the GWAS, eQTL study and the correlation between local GEBV and gene expression, the false discovery rate (FDR) was calculated as *FDR* = *nSign*/(*t* × *nTests*), where *nSign* is the number of sequence variants or correlations with a p-value ≤ *t* and *nTests* the total number of tests, and *t* the significance threshold used.

## Conclusions

Both QTL and eQTL are common and this fact, combined with the long-range LD in cattle, means that a QTL will often be in LD with an eQTL. This situation is difficult to distinguish from an eQTL and a QTL being identical. We used several strategies to distinguish these two possibilities but suspect that they lack enough power to find all cases where the QTL is really an eQTL. Nevertheless, we did identify some cases where the QTL may be identical to an eQTL.

However, it may be that eQTL explain only a small fraction of QTL, and the majority of QTL are influenced by other factors, such as amino acid sequence, splicing of transcripts, post-transcription and post-translational changes.

## Additional files

Additional file 1: Correlations between local GEBV and gene expression. Manhattan plots showing the correlation between local GEBV for milk yield, protein yield, fat percentage, protein percentage and fertility and gene expression. (DOCX 66 kb)

Additional file 2: Association between *FUK* and *DDX19B* expression and fertility. Top left = variance of local GEBV of 250 kb intervals where var.% = variance explained by an interval as percentage of the sum of the variance explained by the non-overlapping intervals that explained the most variance, top right = GWAS for fertility, middle left = −log10(p) of correlations between local GEBV and *FUK* expression, middle right = association between sequence variants and *FUK* expression, bottom left = −log10(p) of correlations between local GEBV and *DDX19B* expression, bottom right = association between sequence variants and *DDX19B* expression. In all graphs, intervals or variants located within intervals with a p_cor(locGEBV,expr)_ ≤ 10^− 5^ are indicated in red (PNG 10 kb)

Additional file 3: Milk yield GWAS and eQTL study results for chromosome 1 around *SLC37A1.* Top left = GWAS for milk yield, top right = variance of GEBV of 250 kb intervals where var.% = variance explained by an interval as percentage of the sum of the variance explained by the non-overlapping intervals that explained the most variance, middle left = *p*-value of correlation between local GEBV in 250 kb intervals and *SLC37A1* expression using milk cells, middle right = p-value of correlation between local GEBV in 250 kb intervals and *SLC37A1* expression using white blood cells, bottom left = eQTL study for *SLC37A1* expression using milk cells, bottom right = eQTL study for *SLC37A1* expression using white blood cells (PNG 10 kb)

Additional file 4: Protein yield GWAS and eQTL study results for chromosome 11 around *PAEP.* Description of data: Top left = GWAS for protein yield, top right = variance of GEBV of 250 kb intervals where var.% = variance explained by an interval as percentage of the sum of the variance explained by the non-overlapping intervals that explained the most variance, bottom left = p-value of correlation between local GEBV in 250 kb intervals and *PAEP* expression using milk cells, bottom right = eQTL study for *PAEP* expression using milk cells. (PNG 7 kb)

## Abbreviations

50 K: Illumina Bovine SNP50 chip
CLPP: Colocalisation posterior probability
DTD: Daughter trait deviations
eQTL: Expression quantitative trait loci
fat: Fat yield
fat%: Fat percentage
FDR: False discovery rate
fert: Fertility
GEBV: Genomic estimated breeding value
GWAS: Genome wide association study
HD: Illumina 800 K BovineHD bead chip
LD: Linkage disequilibrium
MAF: Minor allele frequency
milk: Milk yield
prot: Protein yield
prot%: Protein percentage
QTL: Quantitative trait loci
TD: Trait deviations
