# Supplementary material for: Overlap between eQTL and QTL associated with production traits and fertility in dairy cattle
Source: BMC Genomics. 2019 Apr 15;20:291. doi: 10.1186/s12864-019-5656-7 (PMC6466667; doi:10.1186/s12864-019-5656-7)

**Additional File 1A. Correlations between local GEBV for milk yield and gene expression.** Top = milk cells, bottom = white blood cells, correlations were estimated between 250kb intervals and all genes within 1 Mb of the intervals, y-axis = -log10(p-value of the correlation between local GEBV and gene expression).


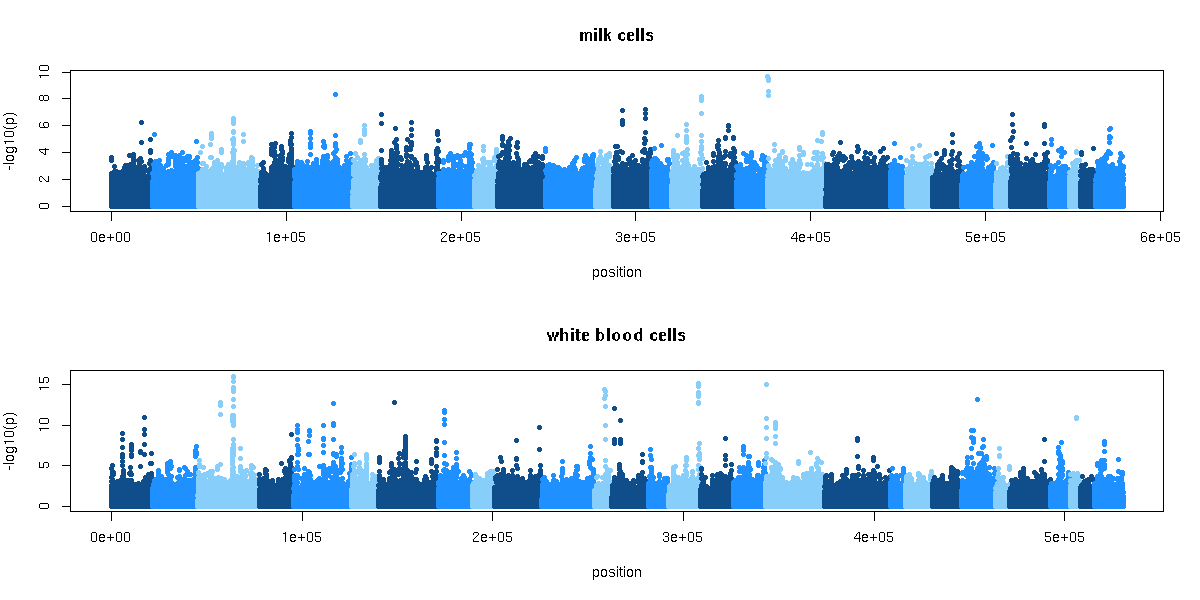


**Additional File 1B. Correlations between local GEBV for protein yield and gene expression.** Top = milk cells, bottom = white blood cells, correlations were estimated between 250kb intervals and all genes within 1 Mb of the intervals, y-axis = -log10(p-value of the correlation between local GEBV and gene expression).


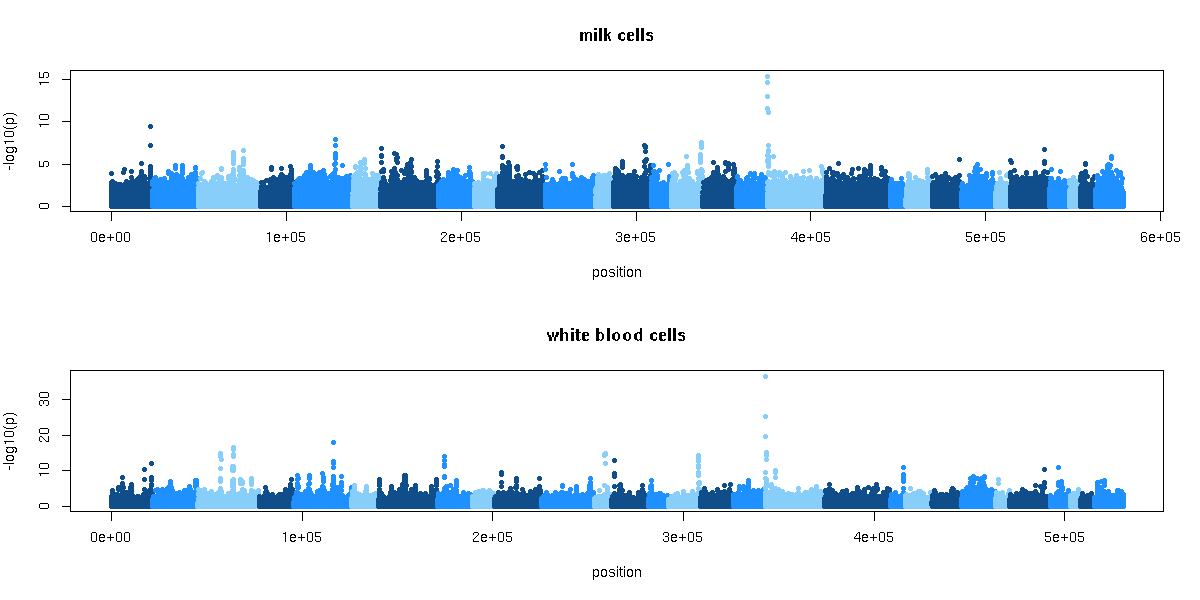


**Additional File 1C**. **Correlations between local GEBV for fat percentage and gene expression.** Top = milk cells, bottom = white blood cells, correlations were estimated between 250kb intervals and all genes within 1 Mb of the intervals, y-axis = -log10(p-value of the correlation between local GEBV and gene expression).


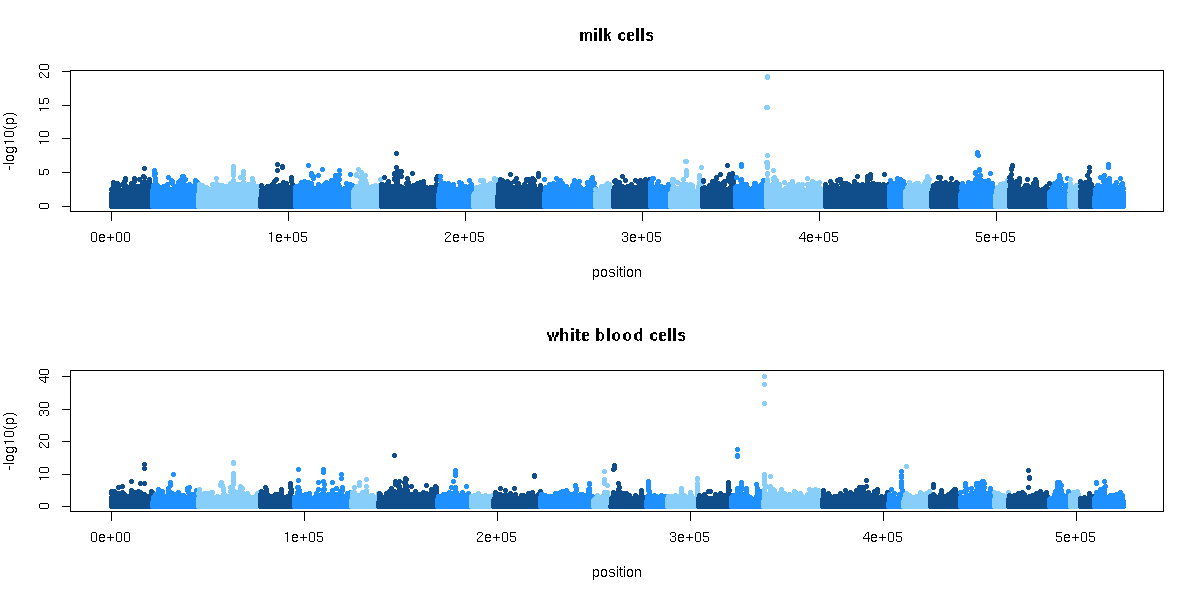


**Additional File 1D. Correlations between local GEBV for protein percentage and gene expression.** Top = milk cells, bottom = white blood cells, correlations were estimated between 250kb intervals and all genes within 1 Mb of the intervals, y-axis = -log10(p-value of the correlation between local GEBV and gene expression).


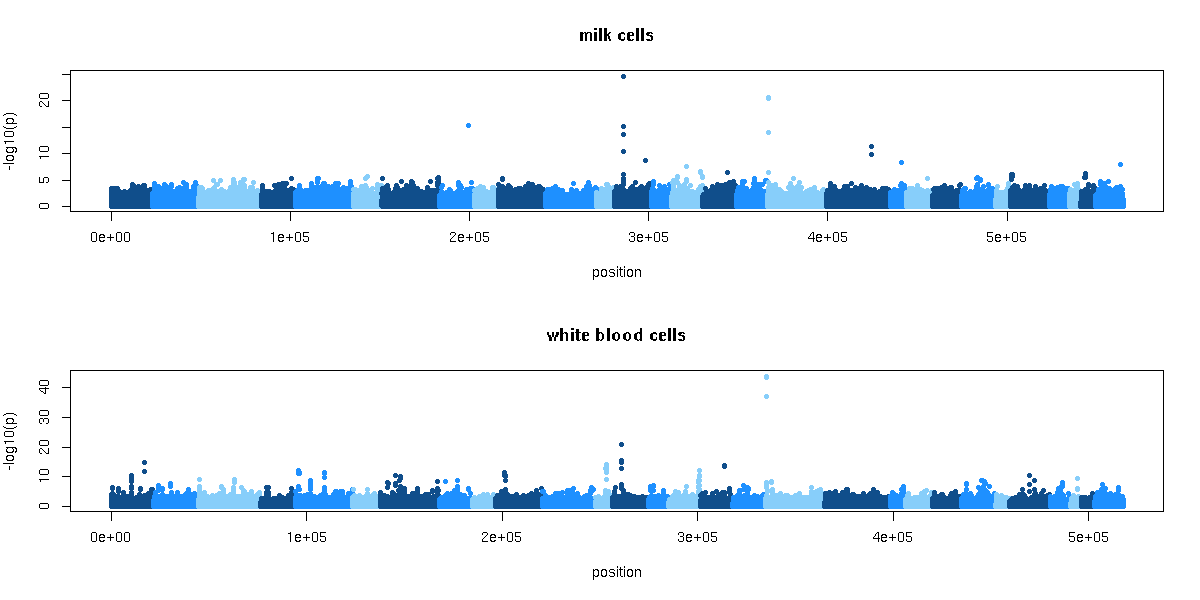


**Additional File 1E. Correlations between local GEBV for fertility and gene expression.** Top = milk cells, bottom = white blood cells, correlations were estimated between 250kb intervals and all genes within 1 Mb of the intervals, y-axis = -log10(p-value of the correlation between local GEBV and gene expression).


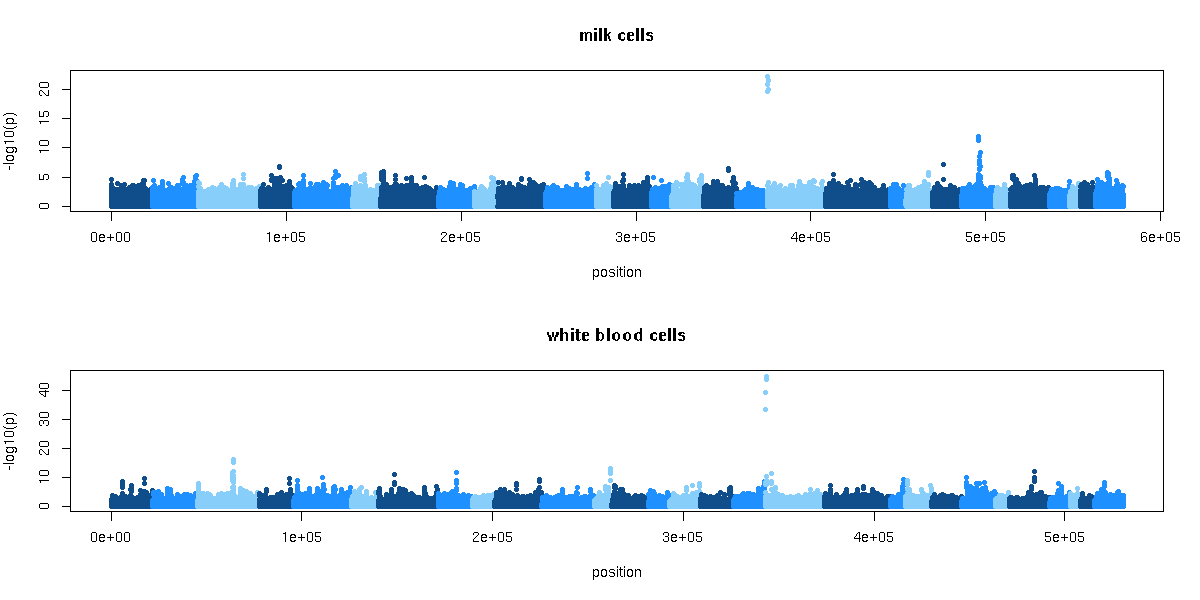

Supplement: Supplementary file 1 — Correlations between local GEBV and gene expression. Manhattan plots showing the correlation between local GEBV for milk yield, protein yield, fat percentage, protein percentage and fertility and gene expression. (DOCX 66 kb) [file 12864_2019_5656_MOESM1_ESM.docx]
