# Supplementary figures and images for: Overlap between eQTL and QTL associated with production traits and fertility in dairy cattle
Source: BMC Genomics. 2019 Apr 15;20:291. doi: 10.1186/s12864-019-5656-7 (PMC6466667; doi:10.1186/s12864-019-5656-7)

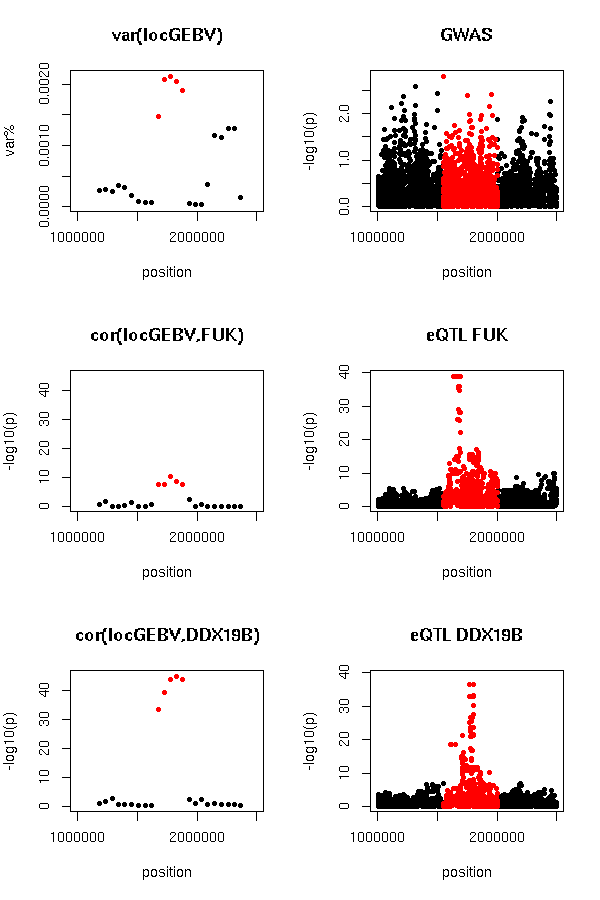

Supplement: Supplementary file 2 — Association between FUK and DDX19B expression and fertility. Top left = variance of local GEBV of 250 kb intervals where var.% = variance explained by an interval as percentage of the sum of the variance explained by the non-overlapping intervals that explained the most variance, top right = GWAS for fertility, middle left = −log10(p) of correlations between local GEBV and FUK expression, middle right = association between sequence variants and FUK expression, bottom left = −log10(p) of correlations between local GEBV and DDX19B expression, bottom right = association between sequence variants and DDX19B expression. In all graphs, intervals or variants located within intervals with a pcor(locGEBV,expr) ≤ 10− 5 are indicated in red (PNG 10 kb) [file 12864_2019_5656_MOESM2_ESM.png]

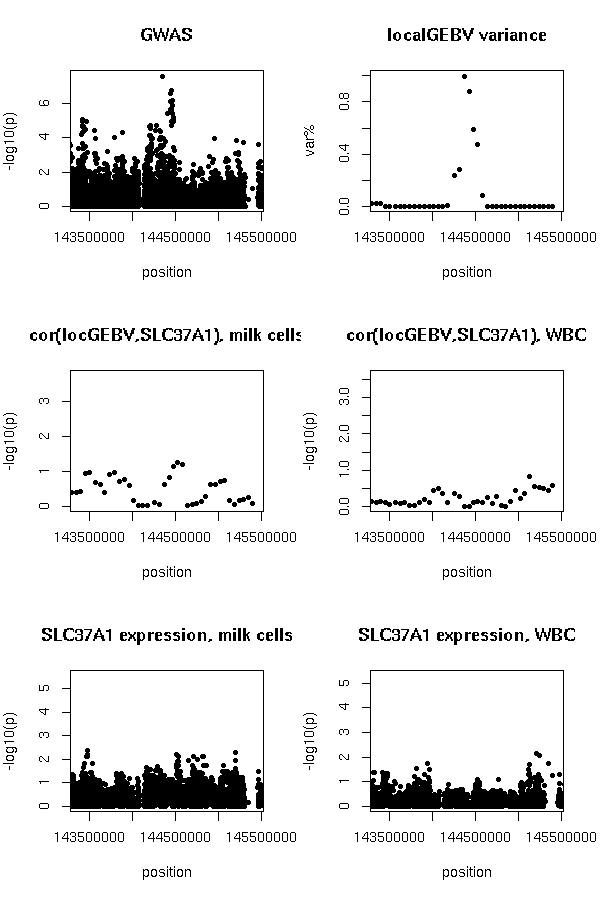

Supplement: Supplementary file 3 — Milk yield GWAS and eQTL study results for chromosome 1 around SLC37A1. Top left = GWAS for milk yield, top right = variance of GEBV of 250 kb intervals where var.% = variance explained by an interval as percentage of the sum of the variance explained by the non-overlapping intervals that explained the most variance, middle left = p-value of correlation between local GEBV in 250 kb intervals and SLC37A1 expression using milk cells, middle right = p-value of correlation between local GEBV in 250 kb intervals and SLC37A1 expression using white blood cells, bottom left = eQTL study for SLC37A1 expression using milk cells, bottom right = eQTL study for SLC37A1 expression using white blood cells (PNG 10 kb) [file 12864_2019_5656_MOESM3_ESM.png]

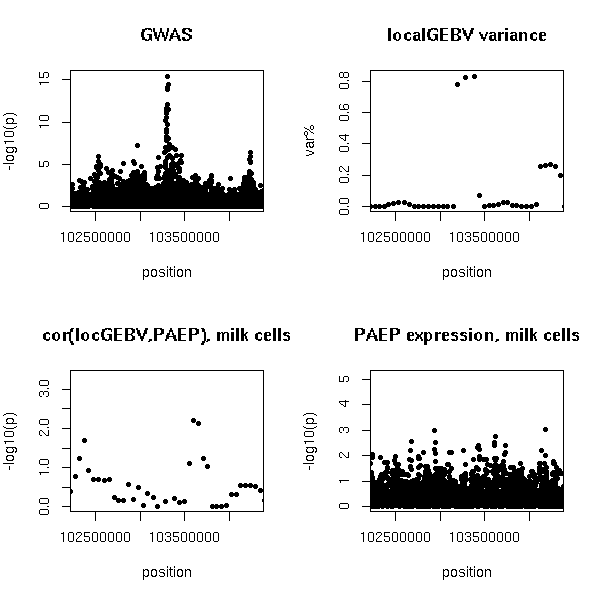

Supplement: Supplementary file 4 — Protein yield GWAS and eQTL study results for chromosome 11 around PAEP. Description of data: Top left = GWAS for protein yield, top right = variance of GEBV of 250 kb intervals where var.% = variance explained by an interval as percentage of the sum of the variance explained by the non-overlapping intervals that explained the most variance, bottom left = p-value of correlation between local GEBV in 250 kb intervals and PAEP expression using milk cells, bottom right = eQTL study for PAEP expression using milk cells. (PNG 7 kb) [file 12864_2019_5656_MOESM4_ESM.png]
